# TUSK: a ubiquitin hydrolase complex modulating surface protein abundance in trypanosomes

**DOI:** 10.3389/fpara.2023.1118284

**Published:** 2023-04-27

**Authors:** Kayo Yamada, Ning Zhang, Farzana K. Yaqub, Martin Zoltner, Mark C. Field

**Affiliations:** ^1^ School of Life Sciences, University of Dundee, Dundee, United Kingdom; ^2^ Department of Parasitology, BIOCEV, Charles University, Vestec, Czechia; ^3^ Biology Centre, Institute of Parasitology, Czech Academy of Sciences, České Budějovice, Czechia

**Keywords:** surface proteins, trafficking, trypanosomes, ubiquitylation, deubiquitinase, cullin complex, drug sensitivity

## Abstract

Control of protein levels is vital to cellular homeostasis, for maintaining a steady state, to coordinate changes during differentiation and other roles. In African trypanosomes surface proteins contribute to immune evasion, drug sensitivity and environmental sensing. The trypanosome surface is dominated by the GPI-anchored variant surface glycoprotein, but additional GPI-anchored and *trans*-membrane domain proteins are present with known roles as nutrient receptors and signal transducers. The evolutionarily conserved deubiquitinase orthologs of Usp7 and Vdu1 in trypanosomes modulate abundance of many surface proteins, including the invariant surface glycoproteins, which have roles in immune evasion and drug sensitivity. Here we identify multiple trypanosome Skp1 paralogs and specifically a divergent paralog SkpZ. Affinity isolation and LCMSMS indicates that SkpZ forms a heterotrimeric complex with TbUsp7 and TbTpr86, a tetratricopeptide-repeat protein. Silencing SkpZ decreases TbUsp7 and TbTpr86 abundance, confirming a direct association. Further, SkpZ knockdown decreases the abundance of multiple *trans*-membrane domain (TMD) proteins but increases GPI-anchored surface protein levels. Hence, a heterotrimeric complex of TbTpr86, TbUsp7 and SkpZ (TUSK) regulates expression levels of a significant cohort of trypanosome surface proteins mediating coordination between TMD and GPI-anchored protein expression levels.

## Introduction

Ubiquitylation is a central process predominantly involved in mediating protein turnover. Post-translational modification by ubiquitin proceeds *via* several steps and depends on the action of ubiquitin ligases that are responsible for transferring ubiquitin to a client protein (Hershko and Ciechanover, 1998). Cullins are a family of scaffold proteins that support E3 ubiquitin ligases, with members present across eukaryotes (Rojas et al., 2017; Nagai et al., 2018). Cullins combine with E3 RING proteins to form cullin-RING ubiquitin ligases (CRLs) with highly diverse roles, most notably targeted protein degradation. CRLs, such as the Skp1-Cul1-Fbox (SCF) complex, target proteins for ubiquitin-mediated destruction and regulate multiple functions including DNA replication, glucose sensing and limb formation in metazoa. The cullin N-terminus is highly variable and, in the SCF complex, interacts with specific adaptor proteins including Skp1 (S-phase kinase-associated protein 1), to bring substrate proteins close to the E3 ligase Rbx1 (Zheng et al., 2002; Goldenberg et al., 2004; Petroski and Deshaies, 2005). Recruitment of combinations of adaptor proteins (F-box proteins in the SCF complex) to the cullin N-terminus are key to diversifying SCF substrate specificity.

Trypanosomiasis is a vector-borne parasitic disease that can be caused by infection by multiple species of trypanosomes (Lukeš et al., 2022). The parasite surface is the principle interface with the host and possesses adaptations crucial to survival. The complexity of this role is reflected in the diversity of surface proteins expressed by the major human pathogens *Trypanosoma brucei and T. cruzi*, where none of the more abundant surface proteins are shared. The *T. brucei* mammalian bloodstream trypomastigote has an efficient endocytic system enabling rapid recycling of surface proteins, antibody clearance and immune evasion, while the majority of the surface is covered by the GPI-anchored variant surface glycoprotein (VSG), although a considerable diversity of additional proteins is also present (Gadelha et al., 2015; Shimogawa et al., 2018). While VSG itself is responsible for antigenic variation and hence immune evasion, several receptors have also been characterized (most recently MacLeod et al., 2022; Makarov et al., 2023) but most surface proteins remain functionally undefined.

The trypanosome surface is organized into subdomains as the proteomes of the cell body, flagellum and flagellar pocket are distinct (Oberholzer et al., 2011; Gadelha et al., 2015). This differential composition extends to internal compartments, with many proteins shared between endosomes and the surface, while other proteins have distinct locations within the endosomal/surface axis (Gadelha et al., 2015). There are likely multiple mechanisms responsible for protein targeting, including *cis*-elements embedded within proteins and post-translational modifications including phosphorylation and ubiquitylation, together with an uncharacterized gating mechanism at the surface (Mussmann et al., 2004; Allen et al., 2007; Gadelha et al., 2009; Emmer et al., 2011; Baker et al., 2012; Graf et al., 2013).

Invariant surface glycoproteins (ISGs) are an extensive superfamily of *T. brucei-*specific type I *trans-*membrane proteins, with large extracellular domains and comparatively short cytoplasmic regions (Allison et al., 2014). ISGs are comparatively abundant and multiple paralogs within the trypanosome genome for each ISG subfamily suggests possible requirements for sequence diversity and/or for high abundance. Significantly, both ISG75 and the closest relative ISG65, have a considerable presence within both endosomes and the surface and are believed to be receptors, with ISG65 demonstrated as a complement component receptor (MacLeod et al., 2022) and ISG75 serving as a xenobiotic receptor (Makarov et al., 2023). ISG65 and ISG75 exhibit considerable structural homology with VSG (Lukeš et al., 2022) and both are ubiquitylated (Chung et al., 2008). There are clear differences between the mechanisms underlying ISG65 and ISG75 targeting and turnover, but they share at least one deubiquitylating (DUB) enzyme in the trypanosome ortholog of USP7, while TbVdu1, a second DUB, impacts only ISG75 (Zoltner et al., 2015). Ubiquitylation is an important regulator of the trypanosome surface but, given the complexity of ubiquitylation systems across the tree of life, it is unclear how these elements act together.

The functions of Skp1 have remained unclear in trypanosomatids (Rojas et al., 2017, Melo do Nascimento et al., 2020), despite the potential for important contributions towards cell cycle progression and differentiation. Here we show that SkpZ, a trypanosomatid-specific Skp1-like protein, forms a heterotimeric complex with TbUsp7 and TbTpr86, a tetratricopeptide-repeat protein, which we designate as the TUSK complex. Unbiased whole cell proteomics demonstrates that TUSK modulates surface protein abundance, suggesting a major role in shaping the trypanosome surface.

## Results

### Multiple SKP1 paralogs in kinetoplastids

**Figure 1:**
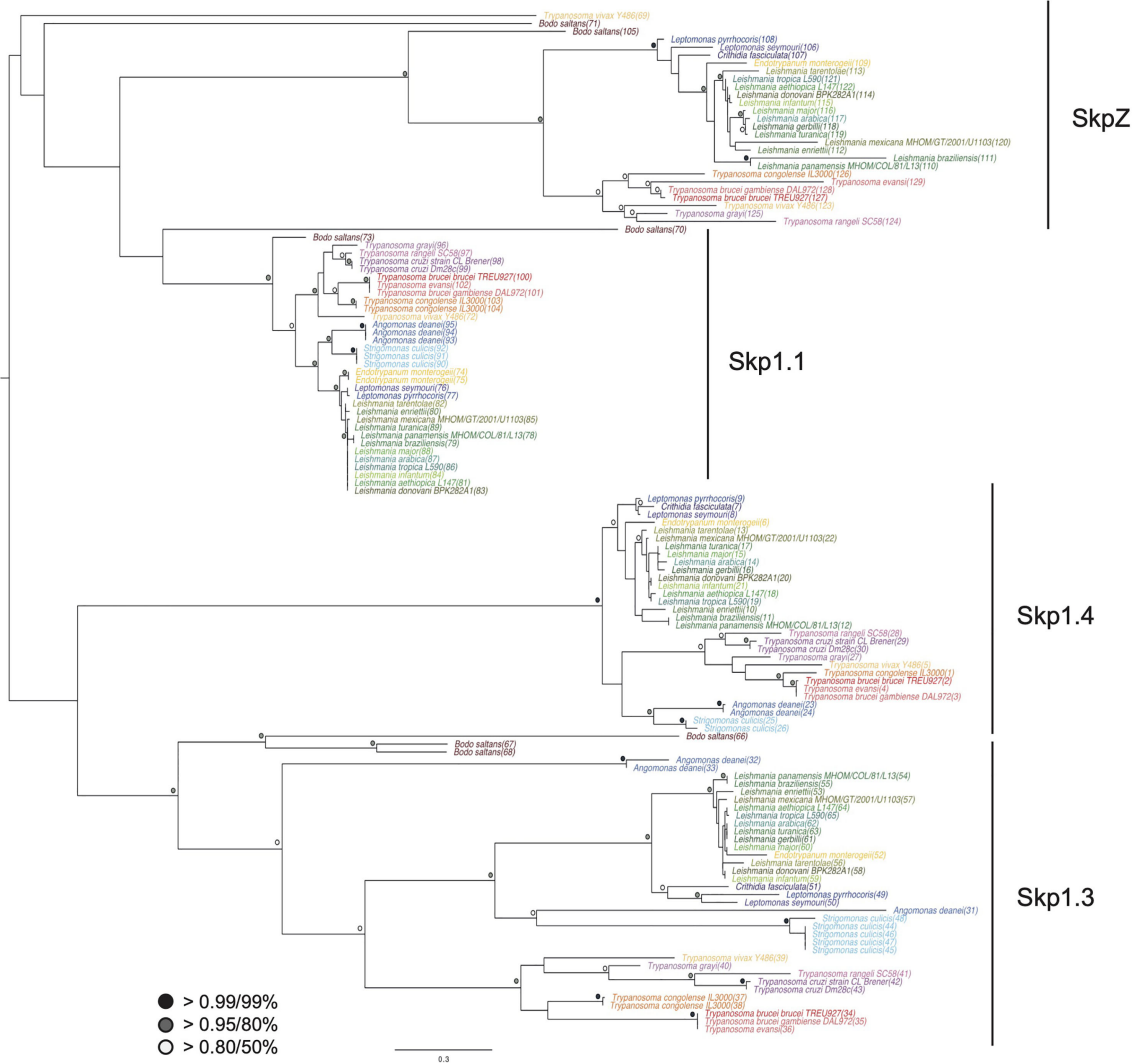
Evolution of trypanosomatid Skp1 proteins. Phylogenetic tree of Skp1 paralogs across kinetoplastid genomes. Trees were constructed using MrBayes and PhyML, with the MrBayes topology shown. Statistical support values are shown as symbols at relevant nodes. Species colors are green/blue for Leishmania and related, blue for insect only parasites, purple for American trypanosomes and red/orange for African trypanosomes. The basal kinetoplastid B. saltans is in tan. Numbers in parentheses following the taxon name refer to accessions given in **Table S1**.

A Skp1-like protein, encoded by Tb927.10.11610, was identified as a suramin sensitivity determinant (Alsford et al., 2012), but to understand the full complexity of Skp1 paralogs in the kinetoplastids we systematically examined the Skp1 family by comparative genomics and phylogenetic analysis. We searched across high quality kinetoplastid genomes and retrieved orthologs from the vast majority. Phylogenetic reconstruction robustly identified four clades of Skp1 paralogs in two clusters. We designated these paralogs as Skp1.1 (Tb927.11.6130), SkpZ (Tb927.10.11610), Skp1.3 (Tb927.11.13330) and Skp1.4 (Tb927.10.14310) (**Figure 1**; **Tables S1**). We propose an updated nomenclature for this family as the presence of multiple paralogs makes designation of a single member as Skp1, the first identified, both likely an inaccurate reflection of lineage-specific expansions within kinetoplastids, as well as the absence of evidence for orthology. TbSkp1.4 is further divergent from both characteristic architectures as above and in possessing an N-terminal extension, such that the protein is considerably larger than other Skp paralogs.

*TbSkpZ is an endosomal protein and interacts with TbUsp7 and an 86kDa Tpr-protein. Homo sapiens* Skp1 localizes to both the nucleus and cytoplasm, but none of the trypanosome Skp1 orthologs apparently share this localization. Significantly, of the Skp1 paralogs, SkpZ uniquely localizes to the endosomal region (Tb927.10.11610), supporting the possibility of a novel function associated with protein trafficking and/or turnover (www.tryptag.org, Dean et al., 2015).

**Figure 2:**
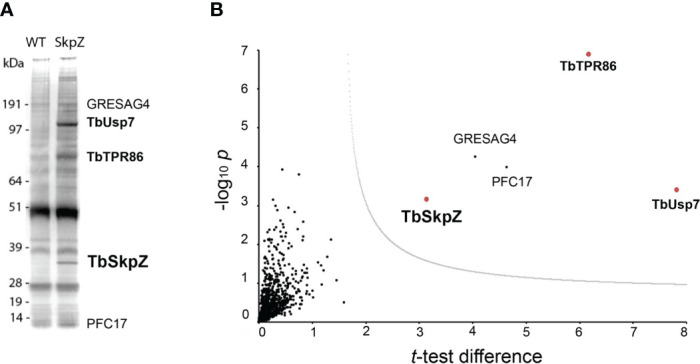
TbSkpZ interacts with TbUsp7 and TbTpr86. **(A)** SDS-PAGE analysis proteins obtained by affinity isolation of 3xHA-tagged TbSkpZ and parental cells (WT) as control. The putative TbUsp7 and TbSkpZ bands, based on estimated molecular weight, were cut from a silver stain gel and individually analyzed by mass spectrometry. **(B)** Volcano plot comparing all proteins coeluted with TbSkpZ with an untagged control elution, and which identifies the TUSK complex. The −log10 t-test p-value was plotted versus the t-test difference (difference between means). The cut-off curve (dotted line) is based on a false discovery rate of 0.05. Members of the Tpr86/Usp7/SkpZ (TUSK) complex are highlighted in bold and red.

To investigate SkpZ further we established an endogenously tagged SkpZ cell line with three copies of the HA-epitope fused at the C-terminus. TbSkpZ HA-tagged cells were harvested and cryo-milled (Obado et al., 2016). We identified conditions for isolation of TbSkpZ together with several additional interacting proteins (**Figure 2**; **Table S3**), which were identified by LCMSMS followed by label-free quantification and statistical analysis in MaxQuant/Perseus (Zoltner et al., 2020). Unexpectedly, TbSkpZ interacted with TbUsp7 rather than cullin complex components, with several additional proteins identified (**Figure 2**). Altogether four proteins, GRESAG4, PFC17, Tb927.11.810 and TbUsp7 were significantly enriched. GRESAG4 has multiple paralogs, is highly abundant and frequently identified in proteomics analyses, which suggests it as a contaminant, while PFC17 (paraflagellar component 17) is a small protein, which increases the potential of non-specific binding and/or detection in mass spectrometry and is, as its name implies, associated with the flagellum. Given no additional evidence for flagellar interaction, we considered PCF17 unlikely a *bona fide* SkpZ partner.

Tb927.11.810 encodes an 86kDa protein with a predicted tetratricopeptide-repeat motif (TPR, IPR011990) located at the center of the protein at residues 246 and 485, and an overlapping pentatricopeptide-repeat region (PPR, IPR002885) at residues 196 to 384, with several experimentally determined phosphorylation sites between residues 84 and 103; we designated this protein TbTpr86. There is evidence for modest upregulation in the mammalian bloodstage compared to insect forms and decreased expression in stumpy forms at the protein level.

Tpr motifs consist of degenerate 34 amino acid repeats and are present in a wide variety of proteins in other organisms, including anaphase-promoting complex subunits cdc16, cdc23 and cdc27, transcription factors, PEX5, the major receptor for peroxisomal matrix protein import and others (Das et al., 1998). Tandem arrays of three to 16 Tpr motifs form scaffolds to mediate protein–protein interactions including assembly of heteromeric and homomeric complexes (Blatch and Lässle, 1999). In *T. brucei* the Tpr motif is retained by the Pex5 ortholog (Gualdrón-López et al., 2013) and predicted for numerous additional proteins.

**Figure 3:**
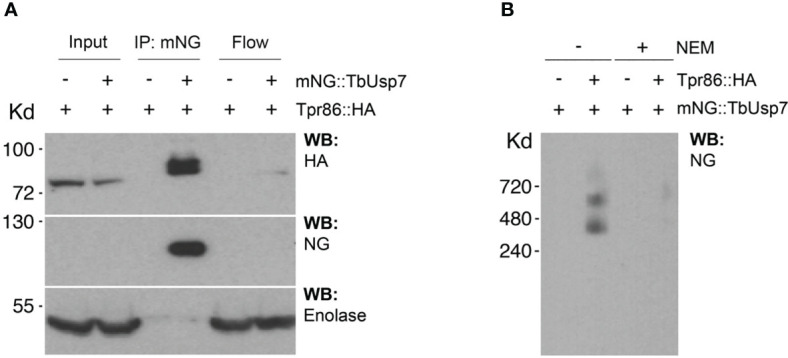
Biochemical characterization of the TUSK complex. **(A)** TbUsp7 co-immunoprecipitated with TbTpr86. Proteins associated with TbUsp7 were purified as indicated via immunoprecipitation. TbUsp7 was detected using anti-mNG antibody and TbTPR86 using anti-HA antibody in whole cell lysates (input), immunoprecipitants (IP), and unbonded fraction (flowthrough). Enolase was blotted as a control for non-specific binding of high abundant cytosolic proteins in the IP. **(B)** BN-PAGE analysis of the TUS complex. Different strains as indicated were lysed with or without N-ethylmaleimide (NEM, 10µM). Whole cell lysates were separated in BN-PAGE and blotted for mNG-TbUSP7 and TbTpr86-HA using respective antibodies.

We designated this complex as TUSK (TbTpr86, TbUsp7 and TbSkpZ) and examined TbUsp7 and TbTpr86 to gain more insight into TUSK activities. First, we attempted to determine the subcellular distributions of both proteins by creating a TbTpr86::3xHA::HYG/BSD::mNG:TbUsp7 line, but localization could not be unambiguously established and is likely an artefact arising from the presence of the tag (**Figure S3**). By contrast, data from TrypTag database (tryptag.org) demonstrates that all three proteins are located across endosomal and possible Golgi complex-associated structures (**Figure S4**), consistent with roles in late exocytic and recycling endosomal compartments. We also used blue native (BN)-PAGE to provide additional evidence of a physical association (**Figure 3**). We observed two high molecular weight species migrating at 400 kDa and 600 kDa in cells where both TbUsp7 and TbTpr86 were tagged and lysates probed with mNeon Green. As the combined molecular weight of the three TUSK subunits is ~200kDa, these data suggest that the detected complexes represent dimers and trimers of subunits, although alternate stoichiometry and/or composition is possible.

### TbSkpZ knockdown inhibits endocytosis and reduces TbUsp7 and Tpr86 protein levels

Cells harboring a stem-loop RNAi construct specific for TbSkpZ were induced with tetracycline. Quantification revealed that knockdown led to enlargement of the flagellar pocket or ‘BigEye’ phenotype. Specifically, 17% of induced cells possessed a BigEye morphology, constituting a six-fold increased frequency compared to uninduced controls. Furthermore, TbSkpZ knockdown resulted in a slight proliferative defect, likely the result of BigEye cells failing to survive.

**Figure 4:**
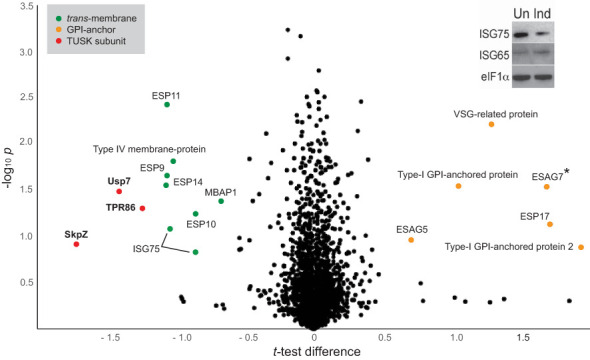
Volcano plot of protein abundance changes following knockdown of TbSkpZ. t-test difference plotted against -log10 transformed t-test p-value. Data points representing protein groups significantly shifted after 48 hours knockdown induction are labeled (for ratio shifts see **Table 1**). Points corresponding to TbUsp7, TbSkpZ and TbTpr86 are plotted in red, GPI-anchored proteins in orange and trans-membrane proteins in green. Note that ESAG7 is annotated with “*” as this soluble protein forms a complex with the GPI-anchored ESAG6. Inset: Silencing TbSkpZ accelerates ISG75 turnover. Cells were silenced by RNAi against TbSkpZ using a tetracyclin (Tet) inducible system for 48 hours and protein levels estimated by Western blotting. The data are a representative example of two experiments, which gave similar results.

A whole cell proteome obtained from SDS-lysates of TbSkpZ-silenced cells was compared with parental cells using stable isotope-labeling by amino acids in culture (SILAC) and LCMSMS. 3904 protein groups were detected, of which 3116 were quantified in both replicates (**Figure 4**; **Table S3**). Significantly, TbSkpZ knockdown reduced levels of TbUsp7 by 38%, ISG75 by ~50%, TbTpr86 by ~42% and SkpZ itself by ~70%, validating the knockdown (**Figure 4**). We were able to demonstrate an ISG75 decrease using Western blotting, providing orthogonal validation for the proteomics data (**Figure 4**, inset). Significantly, TbUsp7 knockdown decreased both ISG75 expression (~40%) and TbTpr86 (~58%) (Zoltner et al., 2015). The impact of TbSkpZ was highly biased towards surface/endosomal membrane proteins as well as proteins potentially associated with endocytosis, specifically VAMP7b SNARE (Tb927.5.3560) (**Table 1**, **Figure 4**) (Venkatesh et al., 2018). The Golgi complex localized Tb927.11.1750 gene product, present only in kinetoplastida, lacks an obvious TMD or GPI-anchor signal or structural homology using Rosetta and AlphaFold (data not shown) but was also decreased.

**Table 1:** Select protein abundance changes in TbSkpZ knockdown cells.

Accession	Annotation	Domains, architectural features	Ratio	-Log10 P-value	Description	
*Tb927.7.6490*	Hypothetical	Predicted GPI	3.99	0.88		**Increased**
*Tb927.7.3260*	ESAG7	GPI, VSG-related fold	3.22	1.53	Transferrin receptor subunit	** **
*Tb927.7.180*	VSG	Predicted GPI, VSG type B-domain	2.44	2.21	VSG-related	
*Tb927.7.6600*	Hypothetical	Predicted GPI	2.07	1.54		
*Tb11.v5.0826*	ESAG5	Tandem bactericidal/permeability-increasing	1.64	0.96	BES11 ESAG5	
Tb927.5.3560	VAMP7B	v-SNARE coiled-coil domain	0.72	0.96	R-SNARE, VAMP7B paralog, endosomal	**Decreased**
Tb927.6.3550	ATPase	TMD, E1-E2 ATPase	0.72	1.83	Phospholipid-translocating P-type ATPase (flippase)	** **
Tb927.9.9410	SLS1	TMD	0.69	0.98	Inositol phosphorylceramide synthase	** **
*Tb927.11.13130*	MBAP1	TMD	0.63	1.38	Membrane bound acid phosphatase 1	** **
*Tb927.2.1700*	ESP10	TMD, EGF-like domain	0.55	1.24	Enriched in surface proteome	** **
*Tb927.5.360*	ISG75	TMD	0.56	0.83	75 kDa invariant surface glycoprotein	** **
*Tb927.5.390*	ISG75	TMD	0.49	1.08	75 kDa invariant surface glycoprotein	** **
Tb927.11.7550	Hypothetical	Two TMD	0.49	1.81	Endosomal location	** **
*Tb927.4.3040*	ESP11	TMD	0.48	2.42	Enriched in surface proteome	** **
*Tb927.9.11480*	ESP9	TMD	0.48	1.65	Enriched in surface proteome	** **
*Tb927.7.470*	ESP14	TMD	0.48	1.56	Enriched in surface proteome	
***Tb927.10.810***	**TbTpr86**	TPR-like	0.42	1.31	Tetratricopeptide-repeat protein	** **
**Tb927.9.14470**	**TbUsp7**	Ubiquitin-specific protease	0.38	1.48	Ubiquitin carboxyl-terminal hydrolase	** **
***Tb927.10.11610***	**SkpZ**	Skp1 dimerisation domain, POZ	0.31	0.92	SkpZ	** **

The ratio of abundance of selected protein groups in control and TbSkpZsilenced cells is shown and data are ranked based on descending ratio. Note that all membrane anchored proteins exhibiting a decrease in abundance possess a trans-membrane domain and increased entries possess GP-anchor linkage. The precise mechanism of anchoring ESAG5 is unclear at present. The accession numbers of the TbSkpZ RNAi target and other TUSK components are in bold. Accession numbers of kinetoplastida-specific proteins are italicized.

**Figure 5:**
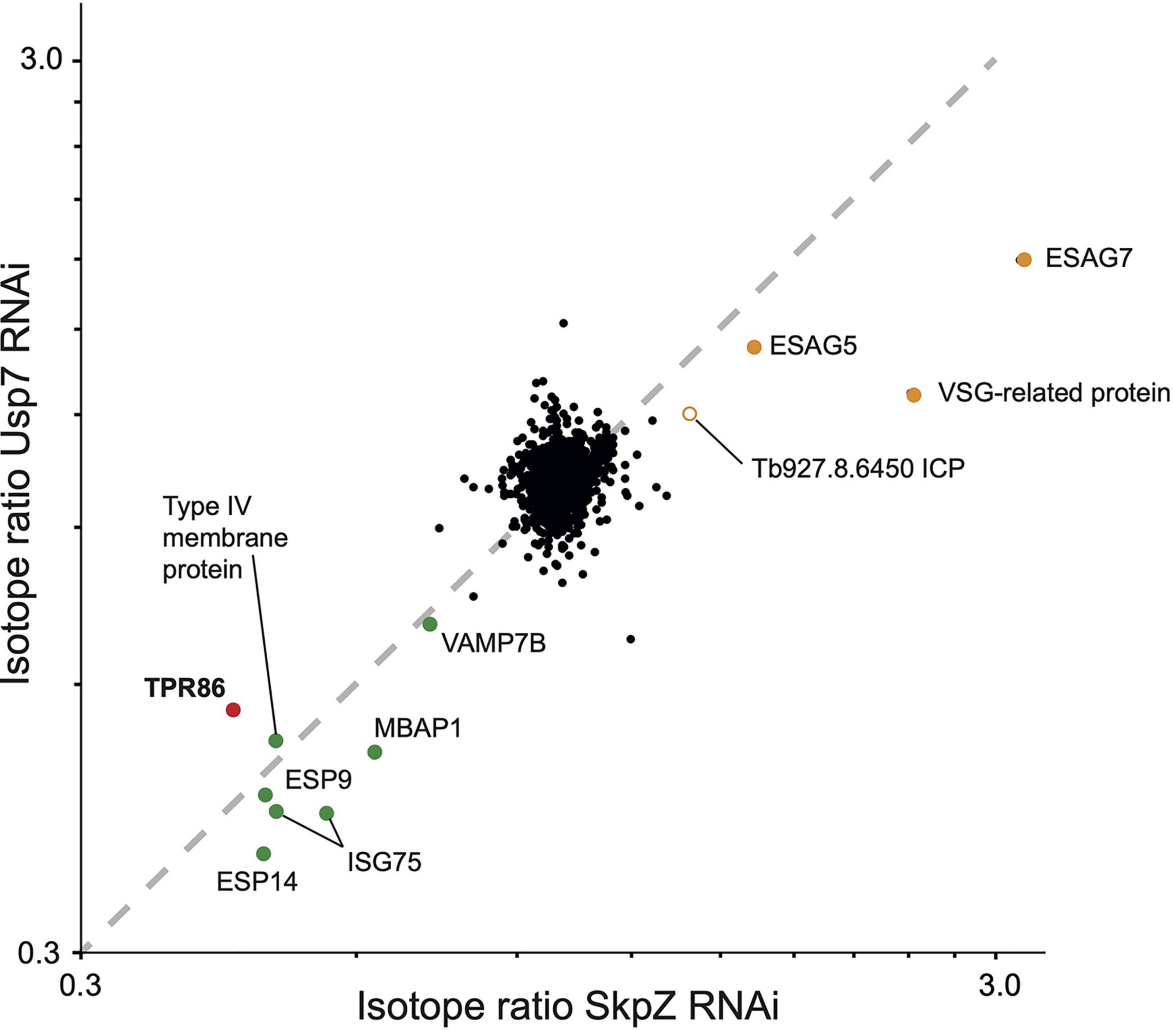
Correlation of proteome changes upon TbSkpZ and TbUsp7 silencing. Abundance shifts after 48 hours silencing of TbSkpZ and TbUsp7 (Zoltner et al., 2015) were plotted against each other. Selected protein groups in the cohorts are labeled and color coded as green; predicted trans-membrane proteins, orange; predicted GPI-anchored, red; TUSK complex component. Note that the proteome analysis of the TbSkpZ RNAi lysate is deeper as extracts were analyzed on a more advanced mass spectrometer accounting for the absence of some proteins from the Usp7 data set.

Most significantly, TbSkpZ silencing impacted surface protein levels depending on their mode of membrane attachment. Specifically, proteins with a predicted TMD were decreased in abundance, while those with a predicted or experimentally determined GPI-anchor were increased. A decrease in abundance is likely due to the absence of DUB activity, *via* loss of TbUsp7, with resulting increased lysosomal delivery of reversibly ubiquitylated proteins. Significantly, these changes are near identical to those previously described for TbUsp7 (**Figure 5**), strongly supporting that both proteins function in the same pathway. Further, many TMD proteins affected are enriched in the *T. brucei* surface-labeled proteome (Gadelha et al., 2015) suggesting that turnover of these surface proteins is also controlled through ubiquitylation and TUSK (**Table 1**).

### TbTpr86 is a pan-kinetoplastid protein

**Figure 6:**
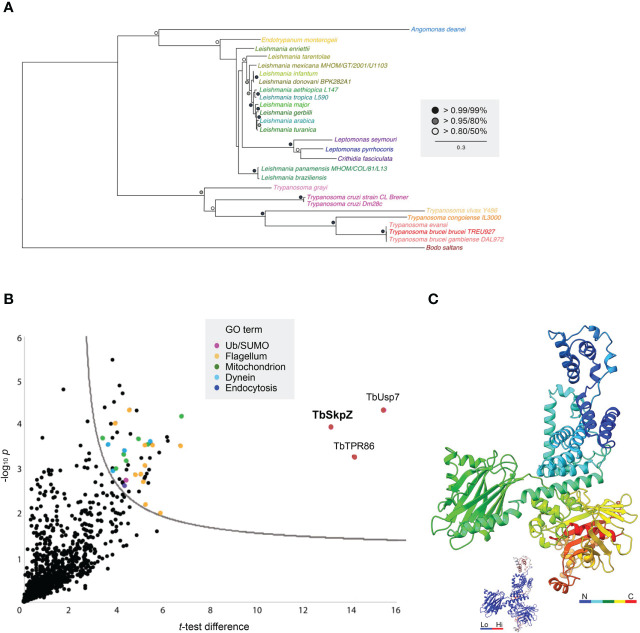
Evolution and interactions of TbTpr86. **(A)** Phylogenetic tree of TbTpr86 paralogs in selected kinetoplastid genomes. Trees were constructed using MrBayes and PhyML, with the MrBayes topology shown. Statistical support values are shown at the relevant nodes. Species colors are arbitrary but consistent throughout all sequence entries and with **Figure 1**. Accession numbers for entries are given in **Table S2**. **(B)** Interactions between TbTpr86 and other proteins in trypanosomes validate the TUSK complex. Affinity isolations from cryo-milled cells with and without a genomically tagged TbTpr86 were performed in three biological replicates. The -log10 transformed t-test p-values were plotted against t-test difference (difference between means). Data points corresponding to TbUsp7, TbSkpZ and TbTpr86 are indicated in red and additional protein groups detected according to the key. Dotted line indicates the confidence limit with a false discovery rate of 0.05. **(C)** Model for TbTpr86 generated with Alphafold, demonstrating predicted structure of the protein. The peptide chain is rainbow-colored as indicated. Inset gives the B-factor values for the structural model, where blue are low and reliable predictions and red are high and uncertain. The corresponding PDB file is available in **Supplementary Data**.

We performed a comparative genomics screen using the TbTpr86 sequence as BLAST query at EuPathDB, NCBI as well as for a HMMER search. The TbTpr86 gene is well conserved and syntenic across the kinetoplastida and extends also present in the bodonids (**Figure 6**; **Table S2**). There is, however, no evidence for orthologs beyond this lineage, suggesting an origin post-speciation from the Euglenida. Molecular modelling, using AlphaFold, indicates that TbTpr86 adopts a complex beta-sheet and a-helical solenoid structure along much of its length; these features could offer multiple interaction platforms for TbSkpZ, TbUsp7 and also recruitment of additional proteins (**Figure 6**).

We established an endogenously HA-tagged TbTpr86 cell line. These cells were harvested and cryo-milled as before. We used essentially the same conditions as developed for the TbSkpZ affinity isolation to identify co-enriching proteins with LCMSMS. Once more, we found strong interactions between TbTpr86, TbUsp7 and TbSkpZ (**Figure 6**), robustly confirming the heterotrimeric TUSK complex. GRESAG4 and PFC17, enriched in the TbSkpZ affinity isolation, were not detected and hence confirmed as contaminants. We only detected additional proteins with significantly lower enrichment, suggesting that additional interactions, if any, were not well retained under the conditions used here.

### TbSkpZ is required to maintain organelle morphology

**Figure 7:**
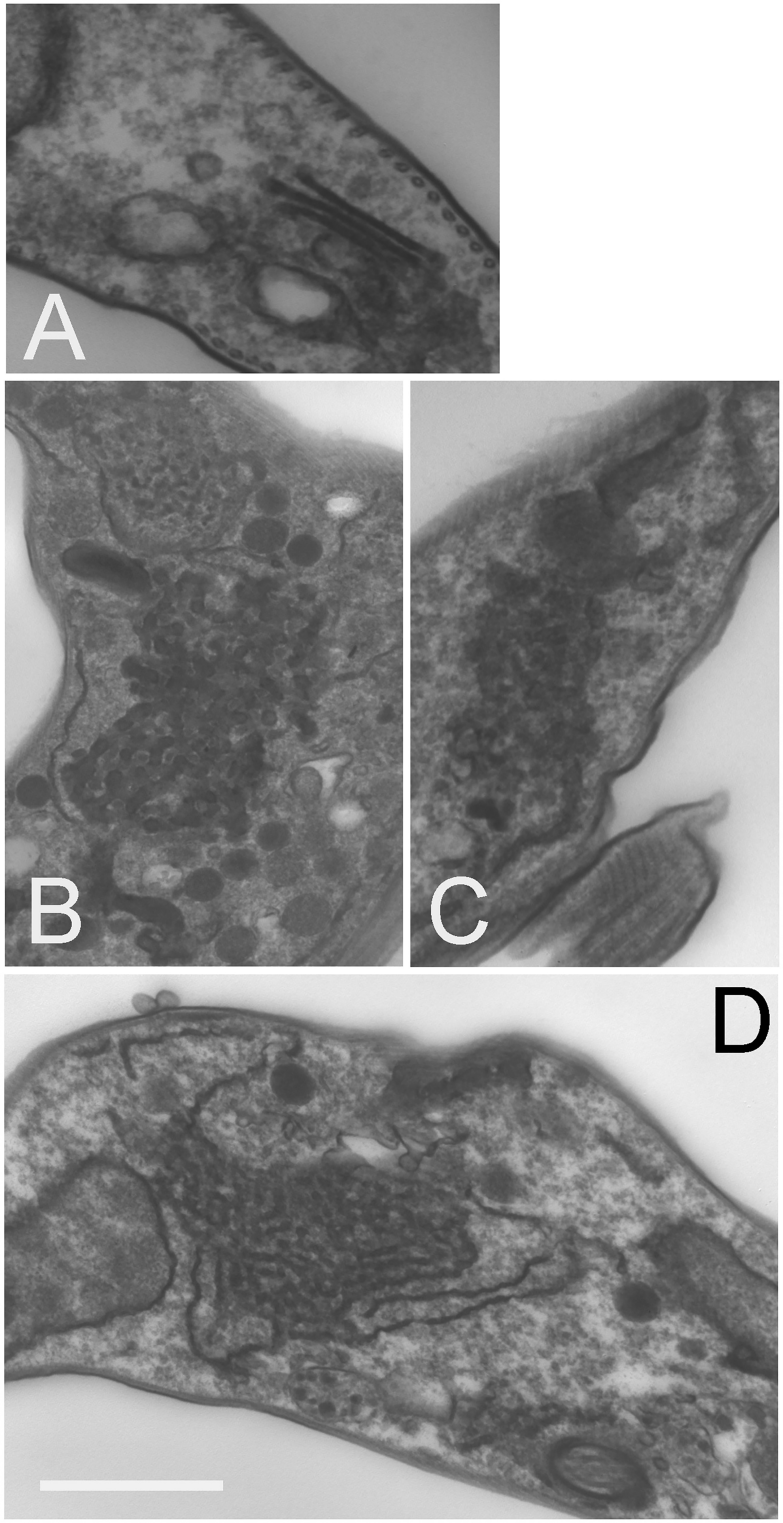
Knockdown of TbSkpZ leads to intracellular membrane hypertrophy. RNAi was induced for 24 hours, fixed and prepared for electron microscopy. **(A)** Uninduced cell showing the Golgi complex region. **(B–D)** Central regions of indices cells showing accumulation of large clusters of electron dense vesicles. In **(D)** the formation of these vesicles from tubular structures is suggested by the aligned membrane structure, and which suggests that these are likely ER and/or Golgi-derived structures. These features were observed in ~30% of sections. Other structures, such as the nucleus, flagellum and cytoskeleton appear unaltered. Scale bar is 1.0um in **(A, D)** and 500nm in **(B, C)**.

Transmission electron microscopy revealed significant accumulation of intracellular vesicles and tubules in TbSkpZ silenced cells, which are extensive. We interpret these structures as likely mainly derived from the anterograde trafficking compartments as they are electron dense, suggesting high protein concentration and specifically the VSG cargo *en route* to the surface (**Figure 7**). These structures appear in the region of the cell usually associated with the Golgi complex and likely recycling endosomal structures (**Figures 7B, C**) and also appear to be associated with the endoplasmic reticulum (**Figure 7D**). We also observed pronounced multivesicular bodies/autophagosomes which is likely suggestive of a need to metabolize excess vesicles (**Figure 7D**), but no obvious impact to other structures, for example the plasma membrane, mitochondrion or the nucleus/nuclear envelope. The contiguous appearance of these novel membrane structures suggests a failure to complete vesicular budding as well as docking. This may be a consequence of the loss of VAMP7b, a protein that is majorly impacted by SkpZ silencing. Regardless of the precise molecular mechanisms, these data indicate a major role for TUSK in maintaining membrane transport and organellar homeostasis.

## Discussion

We describe the heterotrimeric TUSK complex, comprised of the trypanosome ortholog of Usp7, a pan-eukaryotic deubiquitinase, together with kinetoplastida-specific proteins TbSkpZ and TbTpr86. TUSK complex subunit interactions are robustly identified by reciprocal isolations with all three subunits, as well as by an *in trans* impacts to protein abundance following silencing. Significantly, the genes encoding all three proteins can be identified across the kinetoplastida, suggesting that the TUSK complex is present throughout the lineage and arose early following speciation from the euglenids.

TUSK regulates the cell surface proteome, with significant impact on both TMD and GPI-anchored proteins, but affecting these protein cohorts differentially; TMD proteins are downregulated when TUSK subunits are silenced and conversely GPI-anchored proteins are increased in abundance. A decrease in TMD proteins is consistent with the absence of DUB activity removing ubiquitin from endocytosed proteins and hence more efficient targeting to late endosomal compartments. It is possible that the increase in GPI-anchored protein abundance is a secondary consequence; endocytosis is clearly affected by RNAi against TUSK components, and in turn GPI-anchored proteins are upregulated. An impact on several trafficking machinery proteins and MBAP, is also significant as these all participate in endocytic pathways. However, as the GPI-anchored VSG is sorted from other endocytic cargo within sorting endosomes by a clathrin-dependant mechanism, it is also likely that there is a direct impact on the efficiency of this process, in light of the changes to Golgi/endosomal compartment morphology. A modest upregulation in the mammalian bloodstage compared to insect forms may also be a reflection of the higher endocytic activity in the former life stage. Regardless, it is clear that the TUSK complex has major impact on the cell surface proteome and that TUSK is an innovation occurring early within the kinetoplastida. The significant impact on endomembrane compartment morphology and likely reduced consumption of transport vesicles underscores the possible central role of this complex to membrane transport. It is tempting to speculate that this may be associated with the high level of surface GPI-anchored proteins and glycoconjugates in these organisms and that TUSK provides a mechanism to coordinate surface protein cohorts based on their membrane anchor.

The predicted TbTpr86 structure possesses two half beta-barrel-like features supported by α-helices. The overall open structure, with a significant presence of α-helical and unmodeled regions (which may be disordered) suggests that these domains are likely exposed towards the solvent and possibly highly mobile, facilitating binding of additional proteins. If this is the case, it is tempting to speculate that TbTpr86 could act in a manner similar to the flexible cullins which bring substrates close to the Rbx1 E3 ligase, but with this being TbUsp7 DUB in the TUSK complex.

In metazoa USP7 is a high abundance DUB with many roles, including control of the cell cycle *via* p53. USP7 is subject to complex regulatory mechanisms and is itself ubiquitylated and possesses a sophisticated multi-domain architecture involving a self-activation mechanism and several Ubl domains. USP7 can also act as a deSUMOylase (Lecona et al, 2016). Significantly, of many known animal and fungal USP7-interacting proteins, none were identified here and the vast majority are unlikely to be encoded in kinetoplastid genomes, indicating significant diversity in function. Mammalian USP7 forms a cellular switch with E3 ligases to facilitate rapid responses to stimuli, coordination of protein level and multiple functions, including endocytosis (Kessler et al., 2007; Hao et al., 2015; Kim and Sixma, 2017).

**Figure 8:**
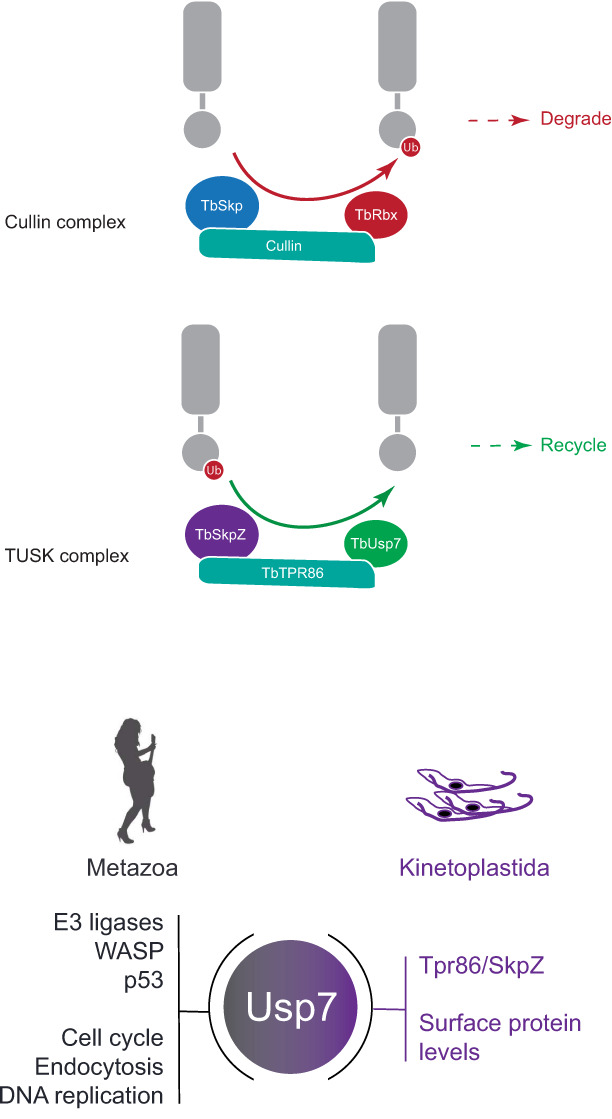
Model for architecture and function of the TUSK complex. Top: Cullin 1 contains TbCullin1 (teal), TbRbx1 (red) and TbSkp1.1 (orange) together with numerous F-box proteins (not shown). Substrates are ubiquitylated (red circle) which targets them for degradation. Bottom: TUSK contains TbTpr86 (teal), TbSkpZ (orange) and TbUsp7 (green). In contrast to Cullin 1, we propose that TUSK is responsible for removal of ubiquitin and which is supported by the decrease in abundance of trans-membrane domain surface proteins following individual knockdown of all TUSK complex subunits.

A rapid switch mechanism seems unlikely in trypanosomes as we did not identify an E3 ligase or evidence for association of ubiquitylation machinery from isolations of members of the TUSK complex. TbTpr86 and TbSkpZ are trypanosome specific, but known roles of Skp1 proteins as substrate adaptors, in association with cullin and Rbx1 containing E3 ligases, suggests a possible similar role. We suggest that the TUSK complex may be a dark-cullin, whereby TbTpr86 provides a scaffold to facilitate TbSkpZ binding substrates and delivering it to TbUsp7 for deubiquitylation, in contrast to the reaction mediated by cullin ligases (**Figure 8**). Hence, while not intimately associated with an E3 ligase as in metazoan organisms, TbUsp7 may none-the-less function in trypanosomes as part of a mechanism providing rapid control of surface protein expression and coordinating levels of GPI-anchored and TMD proteins. Finally, TUSK represents an interesting example of a repurposing of a conserved DUB with lineage-specific components, to augment the function of TbUsp7.

## Methods and materials

### Trypanosome culturing and transfection

Bloodstream form (BSF) trypanosomes were cultured in HMI-11 complete medium (HMI-11 supplemented with 10% fetal bovine serum (FBS) non-heat-inactivated, 100 U/ml penicillin, 100 U/ml streptomycin) at 37°C with 5% CO_2_ in a humid atmosphere, in culture flasks with vented caps. 2T1 cells, a variant of Lister 427 (Alsford and Horn, 2008), were maintained in HMI-11 complete medium in the presence of phleomycin (0.5 μg/ml) and puromycin (1 μg/ml). Following transfection with stem-loop RNAi plasmids or inducible overexpression plasmids, 2T1 cells were maintained in phleomycin (0.5 μg/ml) and hygromycin (2.5 μg/ml) (Alsford and Horn, 2008). Experiments were performed following 48 hours induction with tetracycline (1 μg/ml). Cells were maintained at densities between 1x10^5^ and 2.5x10^6^ cells/ml.

### Recombinant DNA manipulations

*A g*ene-specific fragment of 400–600 bp was amplified with PCR primers designed using RNAit for silencing and cloned into pRPaiSL to generate stem-loop ‘hairpin’ dsRNA to induce RNAi knockdown, using the following primers; TbSkpZSL_F GATCGGGCCCGGTACCATGATTATCGCCCACCAAAC and TbSkpZSL_R GATCTCTAGAGGATCCTTCTCCTCTGACATTCTTCC (Alsford and Horn, 2008). All constructs were verified by standard sequencing methods. TbSkpZ or TbTpr86 was C-terminally tagged using the PCR only tagging plasmids pMOT4H (Oberholzer et al., 2006) or pPOT4H respectively. Primer pairs for DNA amplification: TbSkpZ Forward: TCTTGGCGTGGAGAATGACTTCAAGGCTGAAGAAGAGGCTGAACTCAGGAAAGAGTACGGAAGAATGTCAGAGGAGAAGGGTaccGGGcccCCCctcGAG Reverse: ATCCACTAGTTCTAGAGCGGCCGCCAACATGAGGGTGTGAGGCACACTTGTTTTTGCCGATGTGCGCGTATTCGAGAACCAGGTGTGGCGCGTGTTGACGG

TbTpr86 Forward: TGCGAGGTGCATCTTTACCCTGGGGTTGACGCATTGTTGCTGTCGTTGATGGCTTACTGCCGCTTTAATTGGGAGCAG Reverse: GTAGAACGAAACTAGATAGATAAAGTCACAACACCAGGAGCGCTGCAGTATTAGCTATTATTGTTGTTGCAGCA. For *in situ* tagging, homologous regions encompassing 3’CDS and 3’UTR of individual genes were amplified by PCR together with a HA/mNG tag and Hygromycin/Blasticidin-resistance (HYG/BSD) gene cassette sequences derived from pPOT system (Dean et al., 2014) using the following primers: TbSkpZ::HA::HYG: TCTTGGCGTGGAGAATGACTTCAAGGCTGAAGAAGAGGCTGAACTCAGGAAAGAGTACGGAAGAATGTCAGAGGAGAAGGGTACCGGGCCCCCCCTCGAG; ATCCACTAGTTCTAGAGCGGCCGCCAACATGAGGGTGTGAGGCACACTTGTTTTTGCCGATGTGCGCGTATTCGAGAACCAGGTGTGGCGCGTGTTGACGG; and TPR86::HA::HYG:

TGCGAGGTGCATCTTTACCCTGGGGTTGACGCATTGTTGCTGTCGTTGATGGCTTACTGCCGCTTTAATTGGGAGCAG; GTAGAACGAAACTAGATAGATAAAGTCACAACACCAGGAGCGCTGCAGTATTAGCTATTATTGTTGTTGCAGCA; and BSD::mNG::TbUSP7: 3’CDS: CCTGCAGGTCGACTCTAGAGACTAGTAAGGCAAAATAGAAG; TGCATTATACGGTTTCCAAAATAGCTTTTTTTTAG.3’UTR: GTGGTTCCGGTTCCGGTTCTCTTCAGGTTCTAGGTATTG; AGAACCTTTACTTTCAGAGTCTTCAGGTTCTAGGTATTG. Individual amplicons were introduced into PCF 427 to generate the corresponding strains.

### Transfection

3 x 10^7^ bloodstream-form cells were harvested by centrifugation at 800 x g for 10 min at 4°C. Cells were resuspended in 100 ul of Amaxa human T-cell Nucleofector solution (VPA-1002) at 4°C, mixed with 10 ug (in 5 ul) of linearized plasmid DNA and transferred to electrocuvettes. Transfection was achieved using an Amaxa Nucleofector II with program X-001. Cells were then transferred to tube A containing 30 ml of HMI-9 medium plus any appropriate antibiotic drug for parental cell growth. Serial dilution was performed by transferring 3 ml of cell suspension from tube A into tube B containing 27 ml of HMI-9 medium and repeated again by diluting 3 ml from tube B into tube C. One milliliter aliquots from each dilution were distributed between three 24-well plates and incubated at 37°C. After 6 hours HMI-9 containing antibiotic selection was added to the wells at the desired final concentration. Transformed cells were recovered on days five to six post-transfection.

For procyclic cells, 3 x 10^7^ cells per transfection were harvested at 4°C, washed in cytomix and resuspended in 400 μl cytomix. Electroporation was performed with 5–15 μg of linearized DNA using a Bio-Rad Gene Pulser II (1.5 kV and 25 μF). Cells were transferred to 9.5 ml SDM-79 medium and incubated for 16 hours after which selection antibiotics were added. The cells were then diluted into 96-well microtiter plates. Positive transformants were picked into fresh selective medium 10–15 days post transfection.

### Stable isotype-labeling by amino acids in cell culture labeling

HMI11 for SILAC was prepared essentially as described in (Urbaniak et al., 2012): IMDM depleted of L-Arginine, L-Lysine (Thermo) and 10% dialyzed (10 kDa molecular weight cutoff) foetal bovine serum (Dundee Cell Products) was supplemented with 4 ug/ml folic acid, 110 μg/ml pyruvic acid, 39 μg/ml thymidine, 2.8 μg/ml bathocuproinedisulfonic acid, 182 μg/ml L-cysteine, 13.6 μg/ml hypoxanthine, 200 μM β-mercaptoethanol, 0.5 μg/ml phleomycin and 2.5 μg/ml hygromycin. Finally, either natural isotopic L-Arginine and L-Lysine (HMI11-R_0_K_0_), or L-Arginine ^13^C_6_ and L-Lysine ^4,4,5,5-2^H_4_ (HMI11-R_6_K_4_) (Cambridge Isotope Laboratories) were added at 120 uM and 240 uM respectively. RNAi was induced by addition of 1 μg/ml tetracycline. Equal numbers of induced and uninduced cells, grown in the presence of HMI11-R_0_K_0_ or HMI11-R_6_K_4_ respectively, were mixed, harvested by centrifugation, washed twice with PBS containing complete mini protease inhibitor (Roche) and resuspended in Laemmli SDS running buffer containing 1 mM dithiothreitol and stored at -80°C. TbSkpZ and TbTpr86 RNAi samples were generated in duplicate and triplicate, respectively, with each replicate representing a distinct clone. One label swap was performed in each set of replicates. The heavy isotope incorporation at steady state was determined from one gel slice (60–80 kDa) of a control experiment omitting induction. Samples were sonicated and aliquots containing 5 x 10^6^ cells were separated on a NuPAGE bis-tris 4–12% gradient polyacrylamide gel (Invitrogen) under reducing conditions. The sample lane was divided into eight slices that were excised from Coomassie stained gels, destained and then subjected to tryptic digest and reductive alkylation. Liquid chromatography tandem mass spectrometry (LC-MSMS) was performed on an UltiMate 3000 RSLCnano System (Thermo Scientific) coupled to a Q-Exactive Hybrid Quadrupole-Orbitrap (Thermo Scientific) and mass spectra analyzed using MaxQuant version 1.6 searching the *T. brucei brucei* 927 annotated protein database (release 24) from TriTrypDB. Minimum peptide length was set at six amino acids, isoleucine and leucine were considered indistinguishable and false discovery rates of 0.01 were calculated at the levels of peptides, proteins and modification sites based on the number of hits against the reversed sequence database. SILAC ratios were calculated using only peptides that could be uniquely mapped to a given protein. If the identified peptide sequence set of one protein contained the peptide set of another protein, these two proteins were assigned to the same protein group. Proteomics data have been deposited to the ProteomeXchange Consortium *via* thePRIDE partner repository (Perez-Riverol et al., 2019) with the dataset identifiers PXD021000 (SkpZ RNAi and immunoprecipitation*)* and XD021797 (TbTPR86 immunoprecipitation).

### Immunoprecipitation

Three flat culinary smidgen spoons of cryo-milled protein powders, derived from Skp1-HA, Tpr-HA, or wild type *T. brucei* 427 strain PCF, were dissolved in buffer A (20mM HEPES (pH7.4), 250mM NaCl, 0.01mM CaCl_2_, 1mM MgCl_2_) supplemented with 0.1% Brj58 on ice. The sample was sonicated and centrifuged for 15min, 13000g at 4°C. The supernatant was incubated with anti-HA magnetic beads for two hours at 8°C. After three times washing using buffer A with 0.01% Brij58, the beads were incubated with one times SDS buffer and the supernatant collected for SDS-PAGE as described above for further MS analysis, except samples were run on an OrbiTrap Velos Pro (Thermo Scientific) mass spectrometer. Spectra were processed by label free quantification in Maxquant and subjected to statistical analysis in Perseus (Tyanova et al., 2016) as previously described (Zoltner et al., 2020). All proteomics manipulations were performed using LoBind tubes (Eppendorf) for efficient protein extraction and removal from each tube.

### Preparation of specimens for conventional (resin-embedded) transmission electron microscopy

Cells were pelleted and fixed in 0.1 M Na cacodylate buffer (pH 7.2) containing 4% paraformaldehyde and 2.5% glutaraldehyde for 60 mins, at room temperature. Cells were stained with 1% OsO_4_ with 1.5% sodium ferrocyanide in cacodylate buffer for 60 min followed by 1% tannic acid in 0.1M cacodylate buffer for 1hr and 1% uranyl acetate in acetate buffer for 1hr. Fixates were dehydrated with a 50% to 100% ethanol series followed by 100% propylene oxide. Embedding in Durcupan resin followed standard procedures. Sections were cut on an ultramicrotome at 70-100nm and stained with 3% uranyl acetate followed by Reynolds lead citrate. Grids were imaged on a JEOL 1200EX TEM using an SIS camera.

### Protein electrophoresis and immunoblotting

Proteins were separated by electrophoresis on 12.5% SDS-polyacrylamide gels and then transferred to polyvinylidene difluoride (PVDF) membranes (Immobilon; Millipore) using a wet transfer tank (Hoefer Instruments). Non-specific binding was blocked with Tris-buffered saline with 0.2% Tween-20 (TBST) supplemented with 5% freeze-dried milk and proteins were detected by incubation with primary antibody diluted in TBST with 1% milk for 1 hour at room temperature. Antibodies were used at the following dilutions: mouse monoclonal anti-HA (sc-7392, Santa Cruz) at 1:10,000, rabbit monoclonal anti-myc (7E18, Sigma) at 1:5000, mouse monoclonal anti-v5 (37-7500, Invitrogen) at 1:1000. Following three washes of 10 minutes with TBST, the membrane was incubated in secondary antibody diluted in TBST with 1% milk for 1 hour at room temperature. Commercial secondary anti-rabbit peroxidase-conjugated IgG (A0545, Sigma) and anti-mouse peroxidase-conjugated IgG (A9044, Sigma) were used both at 1:10,000. Detection was by chemiluminescence with luminol (Sigma) on BioMaxMR film (Kodak). Densitometry quantification of relative protein level was achieved using ImageJ software (NIH).

### Blue Native-PAGE

Lysates prepared for Co-IP were analyzed by BN-PAGE in parallel. Samples were diluted with the sample (BN2003, ThermoFisher) and separated under native condition in 4-16% Native PAGE gels (BN1002, ThermoFisher). Proteins were further transferred to PVDF membranes following the standard blotting instruction provided before the detection with designated antibodies. Protein molecular weights were determined with reference to NativeMark™ Unstained Protein Standard (LC0725, ThermoFisher).

### Protein electrophoresis and immunoblotting

Proteins were separated by electrophoresis on 4-12% Bis-Tris NuPAGE gels (NP0322, ThermoFisher) and then transferred to polyvinylidene difluoride (PVDF) membranes (Immobilon; Millipore) using a wet transfer tank (Hoefer Instruments). Non-specific binding was blocked with Tris-buffered saline with 0.05% Tween-20 (TBS-T) supplemented with 5% fat-free milk and proteins were detected by incubation with primary antibody diluted in TBST with 1% milk for 1 hour at room temperature. Antibodies were used at the following dilutions: both anti-ISG65 (rabbit polyclonal from M. Carrington, Cambridge) and anti-ISG75 (rabbit polyclonal from P. Overath, Tubingen) at 1:10,000; anti-EF1α at 1:3000 (mouse monoclonal, CBP-KK1, Sigma); anti-γ-tubulin (mouse monoclonal, MABT163, Millipore) at 1:10,000; anti-mNG (mouse monoclonal, 32F6, ChromoTek) at 1:2,000; anti-HA (rabbit monoclonal, C29F4, CST) at 1:2,000; anti-enolase (rabbit polyclonal from Paul Michels, Louvain) at 1:10,000; an-Ub (mouse monoclonal, P4D1, Santa Cruz) at 1:10,000. Following three washes of 10 minutes with TBS-T, the membrane was incubated in secondary antibody diluted in TBS-T with 5% milk for 1 hour at room temperature. Commercial secondary anti-rabbit peroxidase-conjugated IgG (A0545, Sigma) and anti-mouse peroxidase-conjugated IgG (A9044, Sigma) were used both at 1:10,000. Detection was by chemiluminescence with luminol (Sigma) on BioMaxMR film (Kodak). Densitometry quantification of relative protein level was achieved using ImageJ software (NIH).

### Immunofluorescence

PCF cells were fixed with PBS containing 3.5-3.8% formaldehyde (FA) for 10 mins at room temperature before washing twice with PBS. The fixed cells mounted onto microscopic slices were permeabilized with PBS containing 0.3%Triton X-100 and 5%FBS for 1hr at room temperature. Permeabilized cells were further incubated with corresponding primary antibodies diluted in PBS with 0.3%Triton X-100 and 1%BSA at 4°C overnight. The samples were then washed 3 times with PBS before probing with secondary antibodies conjugated with Alexa flourophores (ThermoFisher). Primary antibodies were prepared as following, anti-HA (rabbit monoclonal, C29F4, CST) at 1:800; anti-mNG (mouse monoclonal, 32F6, ChromoTek) at 1:300; anti-enolase (rabbit polyclonal from Paul Michels, Louvain) at 1:3,000. Images collected from Zeiss Axiovert 200M microscope with a AxioCam MRm camera were analyzed using ImageJ (https://imagej.nih.gov/).

### Bioinformatics

Comparative genomics and phylogenetic reconstructions were performed as described elsewhere. Trees were generated using the NGPhylogeny workflow (https://ngphylogeny.fr). Alphafold predictions for TUSK monomers and complex structures were performed using ChimeraX (Pettersen et al., 2021). All analyses were preformed using default parameters.

## Data availability statement

The datasets presented in this study can be found in online repositories. The names of the repository/repositories and accession number(s) can be found below: https://www.ebi.ac.uk/pride/archive/, PXD021797, PXD021000.

## Author contributions

KY: methodology, investigation, writing - original draft. NZ: investigation, writing - original draft. FY: investigation. MZ: conceptualization, investigation, visualization, supervision, writing - original draft, writing - review and editing. MF: conceptualization, investigation, project administration, funding acquisition, visualization, supervision, writing - original draft, writing - review and editing. All authors contributed to the article and approved the submitted version.

## Supplementary material

Supplementary Figure 1 Alignment of trypanosome Skp1 paralogs. **(A)** Amino acid sequence alignment of four Skp1-like proteins from T. brucei. Protein sequences were retrieved for Trypanosoma brucei TRU927 and aligned using Clustal W. “-” indicates a gap introduced in the alignment, “:” indicates conservative substitution and “*” identity.**(B)** Schematic diagram of TbSkp1 protein paralogs. Skp dimerization domains, as predicted by InterPro (https://www.ebi.ac.uk/interpro/), are overlaid as open boxes.

Supplementary Figure 2 TbSkpZ knockdown perturbs endocytosis. **(A)** Typical case of ‘BigEye’ phenotype, which shows an enlarged flagellar pocket, manifest as a phase-light vacuole at the posterior end of the cell during TbSkpZ RNAi. One hundred cells were analyzed from uninduced or induced cultures of TbSkpZ (closed bar) or TbUsp7 (Zoltner et al., 2015) (open bar) and scored for the appearance of ‘BigEye’ -phenotype. Results represent an average of three independent experiments with error bars denoting the standard error.

Supplementary Figure 3 Validation of epitope integration. SDS-PAGE and Western blot analysis of tagged cell lines. Probed with anti-HA, anti-mNG and γ-tubulin. Whole cell lysates of parental, single and double tagged cells are shown.

Supplementary Figure 4 Localisation of epitope tagged TUSK subunits. Fluorescent micrographs of procyclic form trypanosomes with neon green tagged TUSK subunits as indicated. Neon green is in green and Hoechst 22242 (DNA) in purple. Images are screen grabs from Tryptag (tryptag.org) and cropped for presentation (with permission).

Supplementary Table 1 and 2 Accession numbers for protein sequences included in phylogenetic analysis.

Supplementary Table 3 Proteomics analysis tables for TbSkpZ RNAi SILAC and cryomill affinity isolation of the TUSK complex.

Supplementary Data Sheet 1 Alphafold prediction of the TbTpr86 structure.
