# Supplementary figures and images for: TUSK: a ubiquitin hydrolase complex modulating surface protein abundance in trypanosomes
Source: Front Parasitol. 2023 Apr 27;2:1118284. doi: 10.3389/fpara.2023.1118284 (PMC11732084; doi:10.3389/fpara.2023.1118284)

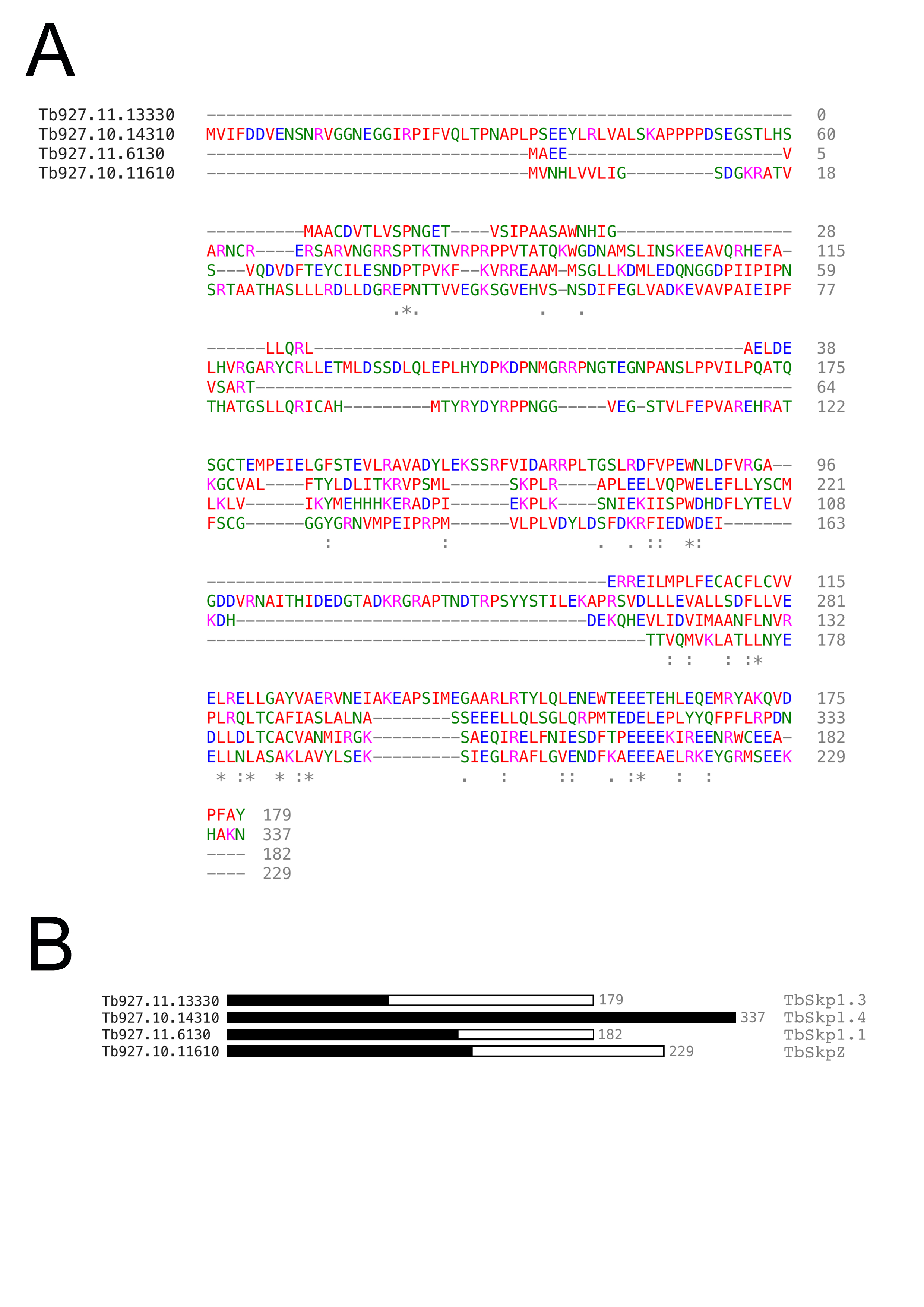

Supplement: Supplementary Figure 1 — Alignment of trypanosome Skp1 paralogs. (A) Amino acid sequence alignment of four Skp1-like proteins from T. brucei. Protein sequences were retrieved for Trypanosoma brucei TRU927 and aligned using Clustal W. “-” indicates a gap introduced in the alignment, “:” indicates conservative substitution and “*” identity.(B) Schematic diagram of TbSkp1 protein paralogs. Skp dimerization domains, as predicted by InterPro (https://www.ebi.ac.uk/interpro/), are overlaid as open boxes. [file Image_1.jpeg]

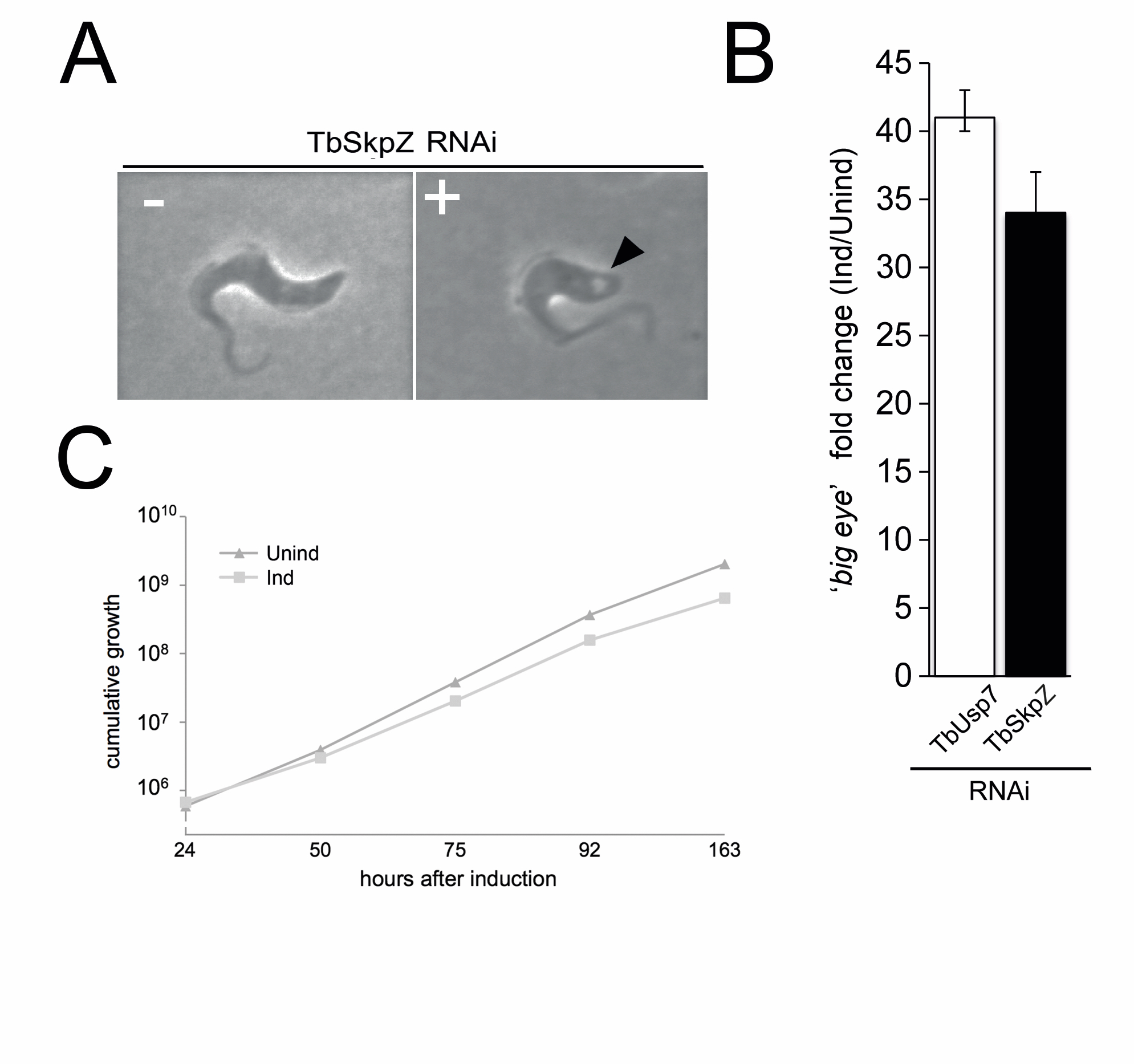

Supplement: Supplementary Figure 2 — TbSkpZ knockdown perturbs endocytosis. (A) Typical case of ‘BigEye’ phenotype, which shows an enlarged flagellar pocket, manifest as a phase-light vacuole at the posterior end of the cell during TbSkpZ RNAi. One hundred cells were analyzed from uninduced or induced cultures of TbSkpZ (closed bar) or TbUsp7 (Zoltner et al., 2015) (open bar) and scored for the appearance of ‘BigEye’ -phenotype. Results represent an average of three independent experiments with error bars denoting the standard error. [file Image_2.jpeg]

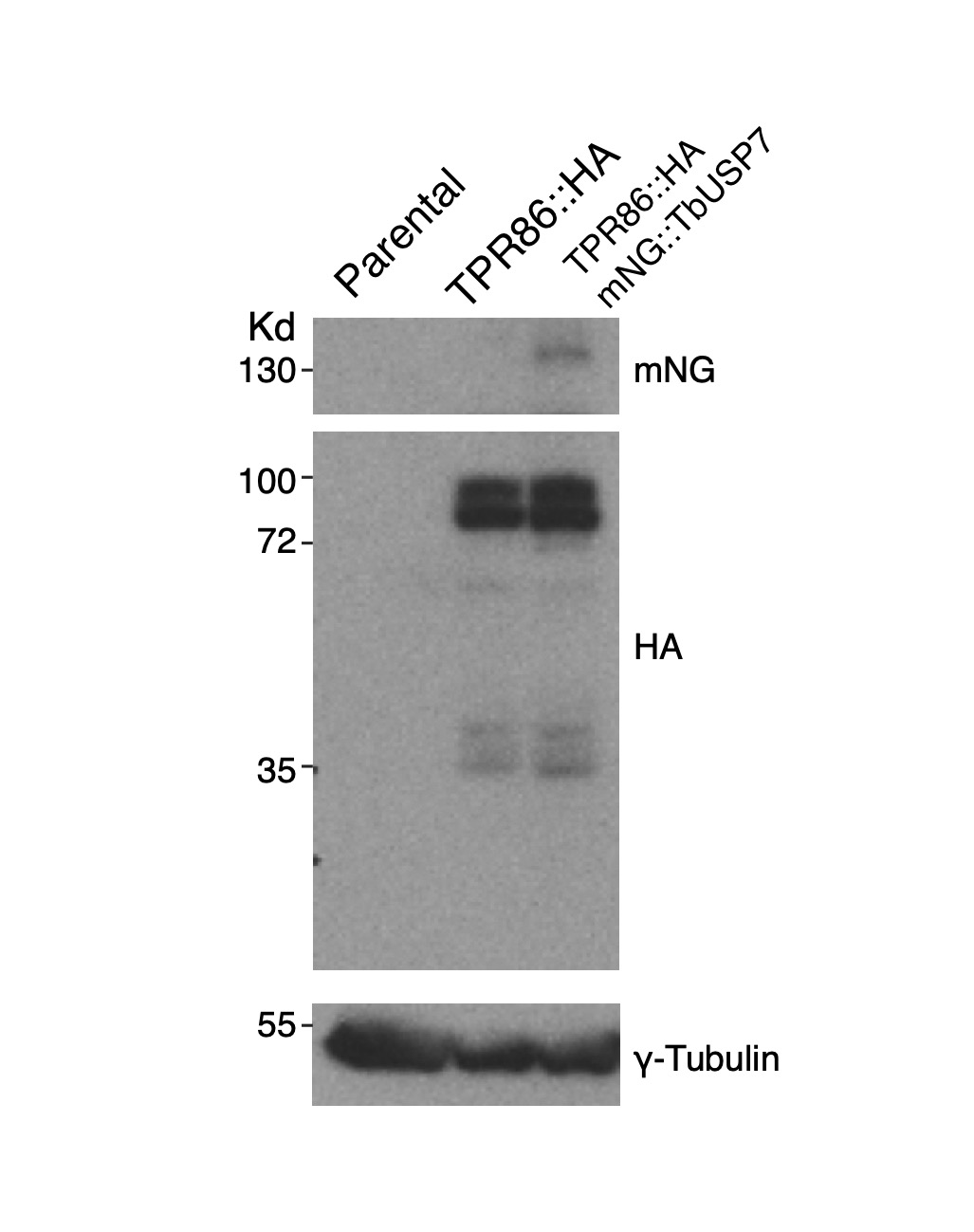

Supplement: Supplementary Figure 3 — Validation of epitope integration. SDS-PAGE and Western blot analysis of tagged cell lines. Probed with anti-HA, anti-mNG and γ-tubulin. Whole cell lysates of parental, single and double tagged cells are shown. [file Image_3.jpeg]

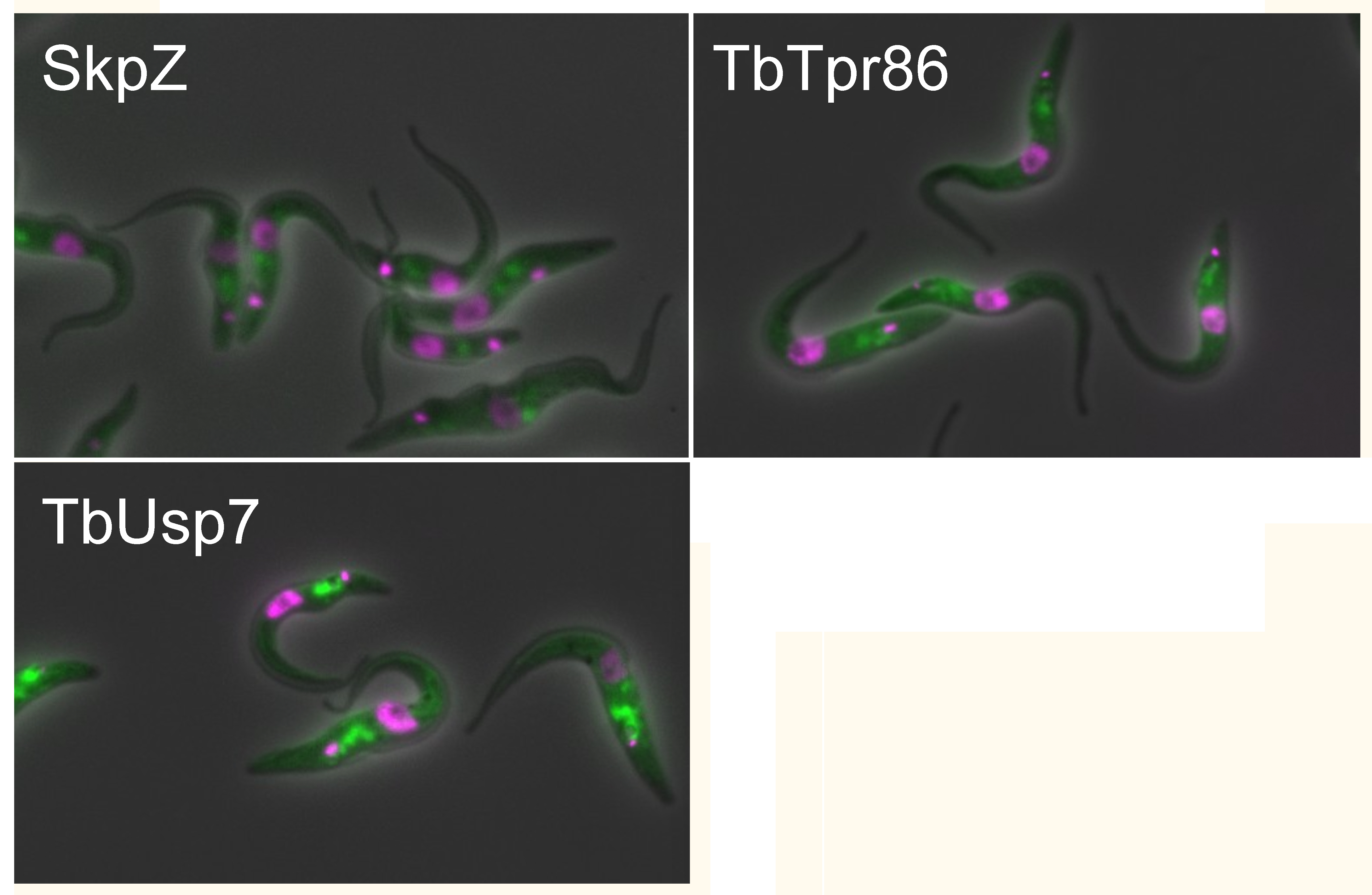

Supplement: Supplementary Figure 4 — Localisation of epitope tagged TUSK subunits. Fluorescent micrographs of procyclic form trypanosomes with neon green tagged TUSK subunits as indicated. Neon green is in green and Hoechst 22242 (DNA) in purple. Images are screen grabs from Tryptag (tryptag.org) and cropped for presentation (with permission). [file Image_4.jpeg]
